# Privacy Protection in AI Transformation Environments: Focusing on Integrated Log System and AHP Scenario Prioritization

**DOI:** 10.3390/s25165181

**Published:** 2025-08-20

**Authors:** Dong-Sung Lim, Sang-Joon Lee

**Affiliations:** 1 School of Cybersecurity, Osan University, Osan 18119, Republic of Korea; seaids77@gmail.com; 2 Interdisciplinary Program of Digital Future Convergence Service, Chonnam National University, Gwangju 61186, Republic of Korea

**Keywords:** privacy protection, security, Integrated log system, ISO/IEC 27001, AHP

## Abstract

Recent advancements in emerging technologies such as IoT and AI have driven digital innovation, while also accelerating the sophistication of cyberattacks and expanding the attack surface. In particular, inter-state cyber warfare, sophisticated ransomware threats, and insider-led personal data breaches have emerged as significant new security risks. In response, this study proposes a Privacy-Aware Integrated Log System model developed to mitigate diverse security threats. By analyzing logs generated from personal information processing systems and security systems, integrated scenarios were derived. These scenarios are designed to defend against various threats, including insider attempts to leak personal data and the evasion of security systems, enabling scenario-based contextual analysis that goes beyond simple event-driven detection. Furthermore, the Analytic Hierarchy Process (AHP) was applied to quantitatively assess the relative importance of each scenario, demonstrating the model’s practical applicability. This approach supports early identification and effective response to personal data breaches, particularly when time and resources are limited by focusing on the top-ranked scenarios based on relative importance. Therefore, this study is significant in that it goes beyond fragmented log analysis to establish a privacy-oriented integrated log system from a holistic perspective, and it further validates its operational efficiency in field applications by conducting an AHP-based relative importance evaluation.

## 1. Introduction

The rapid development of IT technologies such as AI, Big data, IoT, and Blockchain is driving digital innovation, while also rapidly expanding the nature and scale of cyber threats [1,2,3]. Recently, automated hacking techniques utilizing large-scale language model (LLM) are becoming more sophisticated. The attack surface is also increasing due to the expansion of OT and IoT environments, and the activities of cyberwarfare between nations and hacking groups with political objectives are also becoming more active [4,5,6,7]. In addition, the sophistication of ransomware is further complicating the information protection environment of companies and public institutions [8,9,10]. In the past, technical vulnerability exploitation by external hackers was the main focus of security incidents, but recently, cases of customer personal information leaks by authorized internal employees or external service personnel are occurring frequently. For example, in 2023, Tesla leaked personal information such as names and contact information of approximately 75,000 customers by former and current employees [11]. And in the Cash App Investing incident in 2022, a former employee illegally leaked sensitive information of about 8.2 million people [12]. Such cases strongly suggest the need for a system to effectively detect and respond to intentional leaks by insiders.

However, previous studies have often approached security policies from a fragmented and static perspective, and they tend to analyze personal information processing systems and security log frameworks separately, without integration. This approach presents limitations in effectively detecting and responding to sophisticated insider threats and personal data breaches. Although integrated log-based solutions such as Security Information and Event Management (SIEM) are becoming increasingly widespread, there is still a lack of empirical research in real-world environments on the design of concrete threat scenarios and the prioritization of security policies for effective operational use. Accordingly, this study designs a Privacy-Aware Integrated Log System model centered on scenarios that goes beyond simple rule-based detection and is based on the foundation of integrated collection and linked analysis of security logs from heterogeneous systems such as personal information processing systems and security systems, breaking away from the individual log collection method for each internal security solution. In addition, the relative importance of each scenario item was quantitatively analyzed and empirically verified by applying the AHP. Based on the analysis results, if a focused monitoring and detection system is operated centered on key scenarios with high relative importance, it is expected that it will be possible to identify personal information leakage threats early and respond quickly while efficiently distributing operational personnel and resources.

The rest of this article is organized as follows. Section 2 describes related research on integrated log systems, domestic and international security guides such as NIST SP 800-53 and ISO 27001, and personal information leak cases. Section 3 studies proposed models such as unit scenario derivation and integrated scenario derivation methods that can respond to acts that bypass personal information processing systems, security systems, and server systems. Section 4 examines cases where the Privacy-Aware Integrated Log System model is applied, focusing on internal control systems, and verifies the superiority of this model by comparing it with existing operating methods. In addition, the relative importance of scenario items is quantitatively analyzed through AHP to demonstrate operational efficiency. Finally, Section 5 presents conclusions and future works.

## 2. Related Works

This session covers the related theories of this paper, such as the integrated log system and personal information leakage cases. It also describes domestic and international security guidelines, such as NIST SP 800-53, ISO 27001, and ISMS-P, as well as previous studies.

### 2.1. Integrated Log System

Initially, it was a system centered on collecting and storing logs to collect event logs occurring in each security system and activity logs of important business systems and to ensure data integrity required for compliance [13]. Then, it was expanded to Enterprise Security Management (ESM) that simply analyzes logs of perimeter defense system aimed at external perimeter defense such as firewalls, IPS/IDS, and DDoS [14]. Currently, it is changing from basic analysis to three-dimensional correlation analysis based on events of security systems performing internal control and long-term storage logs [15]. Therefore, an integrated log system can be defined as a system that collects and stores events occurring in security systems and IT systems for a long period of time and comprehensively analyzes them to detect and monitor threats in advance. It is also called SIEM from a security perspective and can be viewed as a system that detects internal and external threats.

The integrated log system mainly collects security logs from heterogeneous security systems and has the characteristic of visualizing them through correlation analysis to respond to threats [16,17]. In addition, it is being developed in combination with AI, and many studies are in progress on small LLM and RAG for linking the latest information [18]. Looking into it in more detail, it collects various structured or unstructured logs at high speed from various devices such as perimeter defense systems and business systems that handle internal information. After that, the collected raw data is normalized into a common format through parsing technology and categorized into attack types and managed. Various normalized events contribute to security threat detection through real-time correlation analysis [19,20]. In addition, it is configured to secure security visibility through reporting and dashboard-type visualization. In addition, logs are collected in the form of DB, syslog, snmp, etc. For example, in the case of IPS, event logs are linked to syslog or DB Query and logs such as IP and port that generate malicious code are collected.

### 2.2. Cases of Personal Information Leaks

Looking at the definitions of personal data, the Cambridge Dictionary defines it as “Information held on computers that relates only to you, and that you do not want everyone to know” [21]. If the accession numbers have not yet been obtained at the time of submission, please state that they will be provided during review. They must be provided prior to publication. Personal information includes names, phone numbers, and biometric information, and it is bound to become more important with the development of technologies such as IoT and AI [22]. Therefore, personal information leakage is a security incident that has a significant impact on the reliability and legal responsibility of an organization beyond a simple information leakage. In particular, with the expansion of cloud environments and IT work, the possibility of personal information infringement by insiders or external attackers is increasing. Unauthorized access, mass downloading, and external transmission of personal information are often difficult to detect with individual log systems. Therefore, real-time abnormal behavior detection and post-audit functions based on integrated logs are becoming the core of security monitoring. Accordingly, we examine domestic and international cases of personal data breaches from the perspective of integrated log systems.

In Korea, in 2014, more than 100 million customer personal information from three major card companies was leaked by internal outsourcing employees copying it to a USB [23]. The leaked information included sensitive financial information such as names, resident registration numbers, and account numbers, and it was pointed out that the reasons were insufficient encryption and an integrated log system. As a result, many financial institutions have established an integrated log system. In 2022, a city hall official leaked personal information by accessing it without permission using the authority to search vehicle registrations [24]. This could have been confirmed if an integrated log management system had been applied due to repeated access. In addition, there was a hacking incident at the Korea Research Foundation JAMS system in 2025 [25]. Approximately 120,000 pieces of personal information were leaked through a URL parameter modification attack from an external IP. Meanwhile, in the case of overseas cases, the Capital One hacking incident in the US in 2019 was an incident in which hackers exploited AWS-based web application firewall configuration errors to leak personal information of approximately 100 million customers. They were able to access the instance without access control or separate authentication, allowing them to access a large amount of customer personal information. From the perspective of a service provider, the need for integrated log collection through AWS CloudTrail, combined with secure access control mechanisms, has been highlighted [26]. In the 2018 British Airways personal information leak incident in the UK, malicious code was inserted into the website script, leaking information of approximately 500,000 customers [27]. In the 2014 Benesse Education Group insider leak incident in Japan, an internal employee copied the customer DB to a USB and leaked it externally. If log correlation analysis had been performed for mass queries, downloads, etc., it would have helped with early detection [28]. These domestic and international cases show that the integrated log system is a core infrastructure that goes beyond simple log storage to perform real-time threat detection, abnormal behavior analysis, and legal audit response.

### 2.3. NIST SP 800-53

NIST SP 800-53 is an information system security and privacy control guideline established by the National Institute of Standards and Technology in the United States. It is a standard that structures security controls so that an organization’s information assets can secure the three major security elements of CIA and Privacy [29,30]. The guideline is widely adopted by US federal agencies, private companies, and international organizations, and is designed to selectively customize security controls based on a risk-based approach. It is currently in version 5, and its biggest feature is that it is an extended framework that integrates security controls and privacy controls [31,32]. It structured the previously separate personal information protection guidelines into an independent control family called PT, and it systematically reflected personal information protection principles such as user consent, minimum collection principle, purpose specification, and transparency in the control items. In addition, the system’s impact level is classified into Low, Moderate, and High according to FIPS 199, and it is designed to enable flexible control according to the organizational situation through tailoring based on the Baseline control provided by SP 800-53B.

The entire configuration consists of a total of 20 control families including management, technology, and physical areas, and each control item comprehensively covers the security life cycle such as establishment, implementation, operation, inspection, evaluation, and improvement of the organization’s security policy. In addition, it is possible to maintain consistency between linked systems based on the Open Security Controls Assessment Language (OSCAL) for automated control evaluation. In particular, in terms of personal information leak detection and integrated logs, the Audit and Accountability family provides a basis for tracking abnormal behavior and post-investigation through the creation, storage, and analysis of security audit logs. The System and Information Integrity (SI) family is a detection-centered control that supports the implementation of a real-time leak detection and early response system through the detection of integrity threats, malware, and unauthorized changes within the system. These controls are key elements of the design of personal information leak scenarios based on integrated logs and the advancement of monitoring. Therefore, the theoretical foundation of an excessive access scenario can be linked to the exceeding of the authorized scope of processing under PT-2 and the monitoring items defined in SI-4.

### 2.4. ISO/IEC 27001

ISO/IEC 27001 is an Information Security Management System jointly established by the International Organization for Standardization (ISO) and the International Electrotechnical Commission (IEC) and provides international standards for establishing and operating an information security framework [33,34,35]. This standard mainly tasks the organization to conduct a systematic risk assessment of its information assets, establish security policies based on this, implement control measures, and continuously operate and improve them. In particular, it aims to secure confidentiality, integrity, and availability, and applies the Plan-Do-Check-Act cycle methodology of Dr. Deming of the United States, which is the same as the management system standard system [36,37,38].

The current ISO/IEC 27001 has been reorganized to the 2022 version to take into account the rapid changes in the information security environment. It responds to the latest security threats by reflecting cloud computing, remote work, and personal information protection. The number of controls in Annex A has been reduced from 114 to 93 and has been reorganized into four domains consisting of organizational, people, physical and technological controls. In addition, the consistency between ISO/IEC 27002 and ISO/IEC 27001, which describe the control items in detail, has been strengthened. In addition, a clear purpose description and attribute-based classification system have been introduced to enable flexible customized application of security controls. Meanwhile, from the perspective of personal information leak detection and integrated log-based, several control items play a key role. In the case of Logging, item 8.15, it records structured logs of system and user activities and secures integrity maintenance and auditability, thereby becoming the basis for personal information leak detection. Monitoring Activities item 8.16 supports the identification of abnormal and suspicious activities through real-time and regular monitoring. Additionally, Item 8.23, Information Access Monitoring, analyzes access history to important information to enable early detection of unnecessary or unauthorized access.

### 2.5. ISMS-P

ISMS-P is a certification system for information security and personal information protection management systems in Korea and is operated by the Korea Internet & Security Agency (KISA) based on the Information and Communications Network Act and the Personal Information Protection Act [39]. This system evaluates whether the management system established and operated by an organization to safely protect information assets and personal information is in compliance with the certification standards and officially certifies it.

ISMS-P has been operated as a single certification system since 2018 by integrating the existing information protection management system and personal information protection management system and is suitable for organizations that must simultaneously meet information protection and personal information protection. The certification criteria consist of 101 detailed items in 3 areas. Looking at the contents, there are 16 items for management system establishment and operation, 64 items for protection measures, and 21 major frameworks and detailed items for requirements for each personal information processing stage [40,41]. In particular, it differs from international standards in that it requires protection measures throughout the entire life cycle of personal information, that is, the stages of personal information collection, retention and use, provision, and destruction. ISMS-P adopts a risk-based approach and is designed to flexibly apply controls according to the size of the organization, industry characteristics, and personal information sensitivity, which allows organizations to strengthen their actual security operation capabilities [42,43]. Usually, the scope of ISMS certification includes information systems, locations, and organizations based on information and communication services. On the other hand, the ISMS-P certification scope including personal information additionally includes personal information related to services among the ISMS certification scopes. In other words, the flow of personal information processed in the service and the IT infrastructure processing personal information are added. From an integrated log-based perspective, item 2.9.5, which is a log and access record inspection, regularly checks the access history and activity log of the system and personal information processing system to identify abnormal behavior. And item 2.11.3, which is an abnormal behavior analysis and monitoring, detects and analyzes abnormal access, mass inquiry, and abuse of authority in real time or periodically to prevent personal information leakage in advance. Additionally, the comparison of existing standards and their synthesis with the present study are shown in Table 1.

### 2.6. Related Previous Studies

Looking at related previous studies, Srinivas et al. [44] defined cybersecurity and mentioned that cybersecurity standards and frameworks are necessary for companies and governments to protect important information and infrastructure. Koza [45] stated that the ISO/IEC 27000 family of standards was initially recognized as BS7799 and was introduced as an ISO standard as soon as ISO added it to the ISMS standards and specifically compared and evaluated ISO/IEC 27001 with the NIST cybersecurity framework. And Fonseca-Herrera [46] presented that ISO/IEC 27001 helps organizations protect personal information of employees and customers and manage information risks when applied, but it lacks technical security. Almuhammadi et al. [47] described NIST SP 800 and compared it with ISO/IEC 27001, and Theoharidou et al. [48] explained that insider threats are very important for security management when operating information systems.

Meanwhile, Serckumecka et al. [49] stated that it is expensive to store such data for a long period of time because the number of events collected by SIEM is large, but they mainly focused on firewalls and intrusion detection systems, and did not cover internal control systems, which are personal information processing systems. Eswaran et al. [50] collected endpoint logs and composed scenarios to suggest countermeasures against zero-day attacks, but systematic scenario derivation was insufficient. Cerullo et al. [51] preached the importance of integration for security information and event log management, and mainly focused on the network log perspective, and Fatemi et al. [52] collected and analyzed host-based logs of Windows without using network logs to respond to abnormal signs. This showed the importance of host-based logs, which are internal terminals. Pavlik et al. [53] emphasized the safety of individual resources, even in cloud environments, and suggested utilizing SIEM technology for integrated monitoring and awareness maintenance. Tuyishime et al. [54] presented the initial stages of web firewall security design and cloud-based SIEM construction but explained that it can only be used in small-scale cloud networks. However, it is judged that related studies are fragmentary and focused on general security technologies, and there is a lack of research and efforts to analyze scenario items and relative importance in terms of privacy-aware integrated logs. Ahmad et al. [55] conducted a comprehensive comparative analysis of over 100 existing studies, examining correlation techniques, system architectures, and data sources from multiple perspectives. They proposed one of the most inclusive classification frameworks to date. However, the discussion on integrated scenario-based design was limited, and the analysis lacked sufficient connection to personal data processing systems. Moreover, no empirical case studies involving real-world organizational implementation were provided, and there was little consideration given to prioritization among individual scenarios. Silvestri et al. [56] employed machine learning techniques such as Support Vector Machines (SVM), Bayesian Networks, and Decision Trees to address the issues of excessive alerts and false positives in IDS. However, their approach lacked sufficient integration with behavior-based scenarios related to customer personal data protection, as the focus remained largely on network security logs. Furthermore, research relying on publicly available datasets tends to fall short in capturing the complexities of real-world organizational settings. Wang et al. [57] proposed the addition of noise to mitigate the risk of Membership Inference Attacks that may arise in Secure Inference environments. By introducing noise during post-processing, they demonstrated the ability to defend against the leakage of sensitive data in AI outputs. This approach enables proactive protection against personal information exposure during AI processing. Rajendran et al. [58] focused on unsupervised LLM-based template mining, ensuring that sensitive security logs were processed locally without external exposure. However, their experiments were limited to a specific dataset, highlighting constraints in applying the approach to real-world environments where diverse log types exist.

### 2.7. AHP

Developed by Professor Thomas L. Saaty in the early 1970s, the AHP is a decision-making model that simplifies the entire process to facilitate effective decisions and ultimately guides users toward a final choice [59]. In particular, AHP is a representative multi-criteria decision-making (MCDM) method that has been validated and widely applied across various fields for decades. It is regarded as a highly effective methodology for quantitatively analyzing complex problems and deriving priorities. In AHP, evaluators quantify relative importance through pairwise comparisons. The top level of the hierarchy defines the overall decision goal, followed by evaluation criteria that influence the goal. These criteria can be further broken down into multiple levels of sub-criteria. Pairwise comparisons are conducted by hierarchical level based on survey data. Relative importance among criteria is then estimated from the resulting pairwise comparison matrix. The consistency of the judgments is evaluated using the Consistency Ratio. A Consistency Ratio value within 10% is considered to indicate acceptable reliability.

## 3. Methods

### 3.1. Privacy-Aware Integrated Log System Model

**Figure 1:**
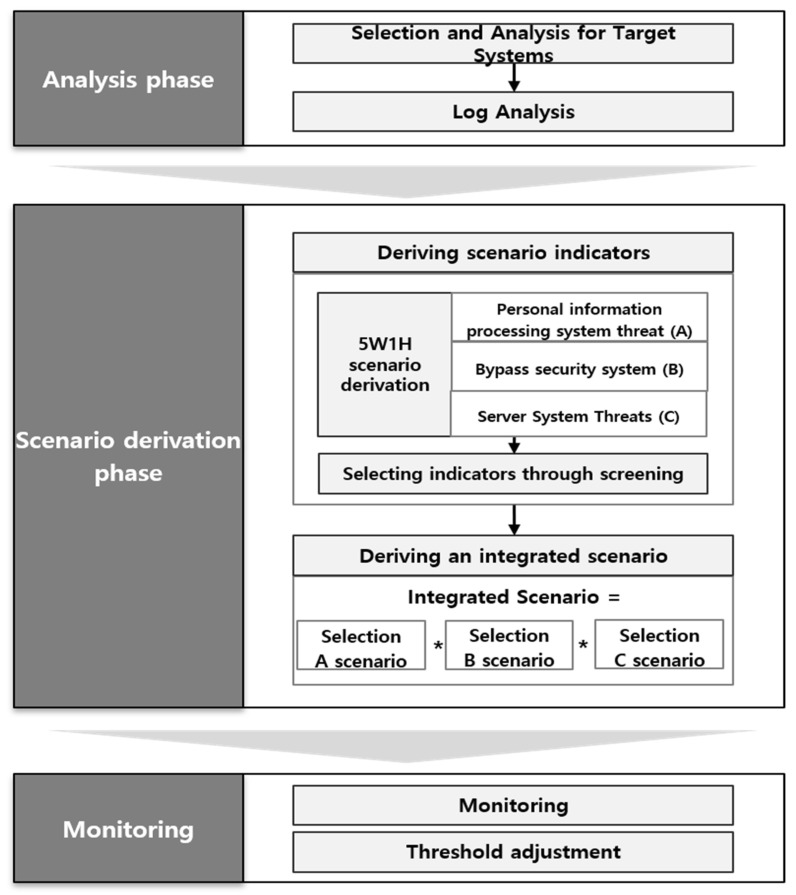
Privacy-Aware Integrated Log System Model.

At a time when digital transformation is taking place due to IoT, 5G, AI, etc., and internal customer personal information leaks are becoming a major issue, an integrated log system in terms of privacy is receiving attention again. In particular, in a situation where internal personal information is leaked due to various attacks such as APT and malware, security systems that are distributed and operated individually in specific information leak response areas have limitations, and a method that can organically link and respond in an integrated manner is needed. In this study, we propose a scenario-centered Privacy-Aware Integrated Log System model that utilizes 5W1H to effectively detect and respond to internal personal information leaks. We developed this model by analyzing domestic and foreign security guides, personal information leak cases, etc., in addition to theoretical examinations that have been previously studied. The contents are as shown in Figure 1.

This research model consists of an analysis stage and a derivation stage of unit scenarios for personal information processing systems, security systems, and server systems bypass. After that, it consists of an integrated scenario derivation stage that integrates the unit scenarios, and a monitoring stage to secure security visibility.

### 3.2. Research Model Step Description

#### 3.2.1. Analysis Phase

To prevent personal information leakage, first select the target systems to be linked and analyze the logs generated by those systems in detail to establish a basis for valid scenario operation.

1. Selection and Analysis of Target Systems; Most companies operate various systems such as PCs, servers, security equipment, network equipment, and IoT devices. Among these infrastructures, it is important to determine and identify key assets for internal control. In order to collect valid logs, they are identified as security systems such as DRM, access control, and PC security, and personal information processing systems such as customer information systems and recruitment systems that have customer information. They are also classified as server systems in terms of the OS that help the personal information processing service operate. The selected systems must determine the IP, linkage cycle, and linkage method for log collection. In particular, information that needs to be identified may be added depending on which linkage method, such as DB, syslog, or File, is selected. For example, in the case of the DB linkage method, it can be the DBMS type, DB Port, DB name, and linkage table. In addition, when considering security, it is necessary to create a separate DB account with only query authority rather than using the existing DB account to collect security logs.
2. Analysis of collected logs; After identifying the systems to be linked, it is important to analyze their logs in detail to determine both the logs currently being collected and those that are critical for addressing personal information protection.

**Figure 2:**
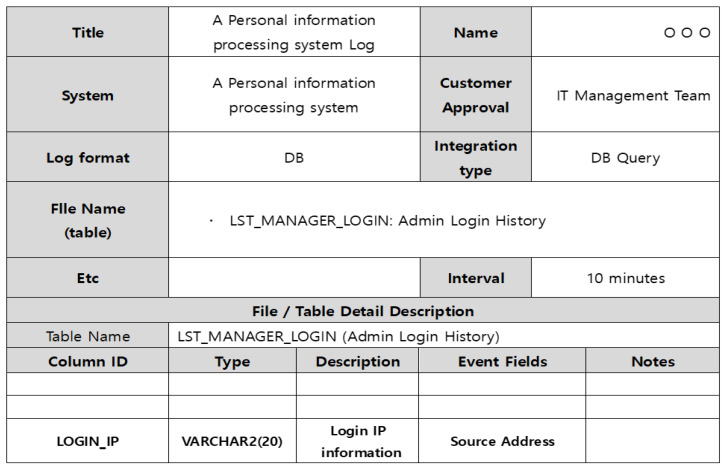
Example of analyzing collected logs for integrated systems.

A specific internal control scenario is derived, but the corresponding logs are not collected, and the corresponding scenario may not be valid because the event does not occur during actual operation. In particular, in the case of a DB linkage method, the schema structure such as the table name containing important internal information and the column name, type, and size of the table must be identified. Through this information, the system linkage and valid logs can be accurately collected. Figure 2 is an analysis of the administrator login history table for a specific personal information system. The IP information accessing the personal information system is stored in the 20-byte varchar2 Login IP column of this specific table. Therefore, operators can use the access login information as important information for controlling internal personal information leakage.

#### 3.2.2. Unit Scenario

An overview of the derivation flow of core unit scenarios is as follows. First, unit scenarios are derived from various log sources such as personal information processing systems, security systems, and servers, based on the 5W1H perspective. A unit scenario refers to a single-condition scenario tailored to a specific threat requirement. These unit scenarios can be selected as core unit scenarios through weighted evaluation based on criteria such as Validity, Risk Relevance, and Frequency. The weight serves as a decision-making factor in prioritizing core scenarios. The selected core unit scenarios are designed so that when a specific threshold value is exceeded during operation, the behavior is considered abnormal, and the corresponding alerts are triggered. The threshold value is a reference metric used to determine whether the unit scenario exhibits anomalous behavior.

**Figure 3:**
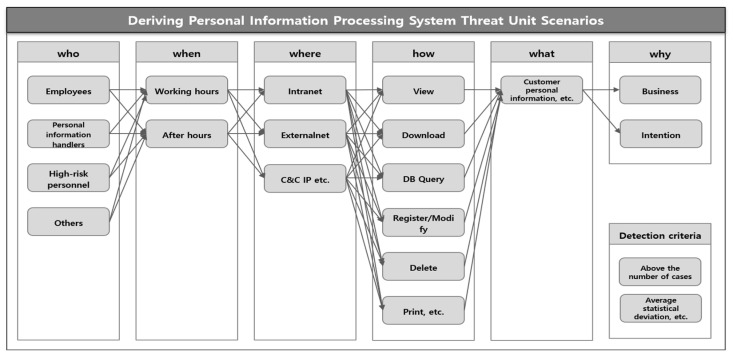
Method for deriving a personal information processing system unit scenario.

**Figure 4:**
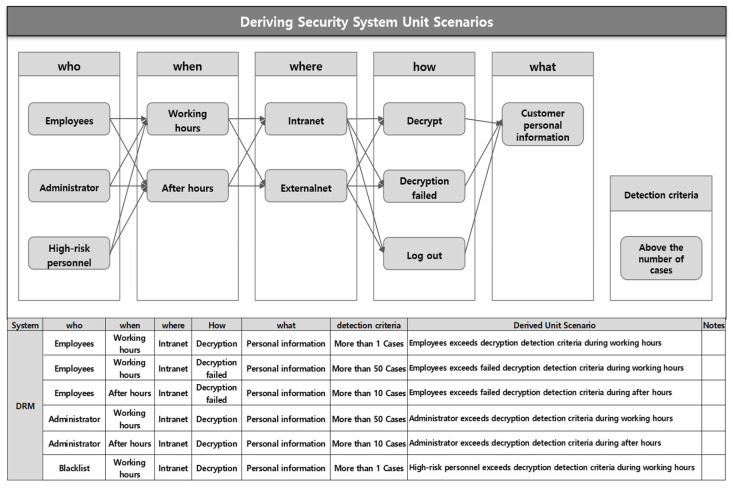
Deriving a security system bypass response scenario.

1. Personal Information Processing System Threat Scenario: In order to respond to threats that may leak important information by illegally accessing personal information processing systems, a unit scenario can be created from the 5W1H perspective. Looking at the six principles of the scenario perspective, there are the Who aspects, such as general employees, retirees, DB operators, DB developers, personal information handlers, contract workers, and Blacklist. There can be the When aspects, such as working hours, after hours, public holidays, and vacations, and there can be the Where aspects, such as the headquarters network, overseas networks, the Internet, and C&C IP. In the personal information processing system, there is the What aspect of personal information documents, important documents, etc. And the How aspect of viewing, downloading, and printing the relevant important information can be categorized into whether or not it was performed. In addition, the relevant action can be classified into the why aspect, such as mistake, work, or intention. Here, we can detect unit scenarios when they exceed the detection criteria through thresholds. The threshold can be measured through criteria such as the number of cases exceeding 1000, the top 5%, or deviations from existing regular patterns. For example, a unit scenario can be derived that can detect an action by a person scheduled to retire who handles personal information in a CRM system that has customer information to perform more than 10,000 queries on a DB table containing customer information at the company on Saturday at 8 PM. This threat scenario can be judged as a possibility of customer information leakage, not work-related. Figure 3 shows the method for deriving a personal information processing system unit scenario.

2. Security System Bypass Response Scenario: These are unit scenarios derived from security systems operated for personal data protection and internal security control. Personal information handlers, authorized users, and blacklists are the Who aspect. Important documents, customer personal information, etc., are the What aspect. Decryption of encrypted documents, external leakage through webmail, etc., can create a security solution bypass control unit scenario based on the How aspect. Figure 4 is a scenario derivation model for detecting illegal activities that attempt to bypass the DRM security system, which is a document security.

In particular, in the security system bypass response scenario, the How aspect can correspond to the main function of the preventive control of each security system. For example, in the document security system DRM, a bypass scenario can be derived in the case where a personal information handler completely decrypts documents containing personal information of multiple customers outside of business hours. In other words, the scenario can be composed around the How aspect of the encryption and decryption functions, which are the main control functions of the document security system.

**Figure 5:**
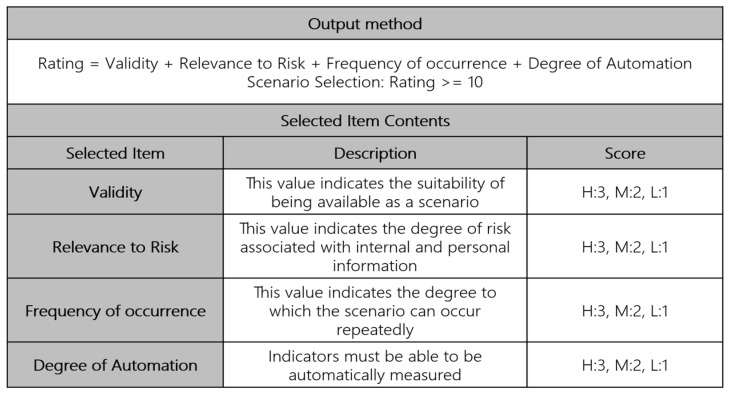
Scenario selection calculation.

3. Server System Threat Scenarios: In this stage, we derive unit scenarios that may pose threats to Linux server systems storing personal information. The who may include system administrators, external attackers attempting to seize root privileges, or internal users. The What aspect corresponds to DB files where personal information is stored, system access logs, etc. The How aspect can appear in the form of abnormal repeated access from specific internal and external IPs, dictionary attacks using weak passwords, and manipulation of audit logs such as audit.log and auth.log after seizing normal administrator authority. Therefore, scenarios can be created based on server system bypass behaviors. For example, multiple SSH connection attempts within a short period of time, root privilege theft and login attempt from a specific IP, and whether log deletion commands are performed are analyzed in conjunction. Through this, scenario design is possible to detect bypass attempts early.
4. Selecting Key Risk Scenarios: Key risk scenarios can be selected by evaluating the scenarios derived from threats to personal information processing systems, security system bypass, and server system bypass based on validity and relevance to risk. The key risk scenario selection items are scored based on validity, relevance to risk, frequency of occurrence, and degree of automation. High is 3, middle is 2, and low is 1, and those scoring 10 or more are selected and classified as key risk scenarios. Figure 5 illustrates these.

The validity and relevance to risk related to the selected items are quantified according to the organization’s security management guidelines and characteristics, and frequency of occurrence and the degree of automation are calculated and adjusted based on the average value after monitoring for a certain period of time. For example, a scenario in which a general employee of a specific company’s security guidelines leaks important documents without decryption authority has a low validity score. If the average number of decryptions is 50 after monitoring for a certain period of time, the frequency and automation-related High can be scored as 110% of the average value, and Low can be scored as 90%.

#### 3.2.3. Integrated Scenario

An integrated scenario can be derived by integrating a personal information processing system threat scenario, a security system bypass response scenario, and a server system threat scenario. The output of the integrated scenario is expressed through combination rules that link each unit scenario using AND conditions. That is, it describes situations where multiple scenarios are simultaneously satisfied at a specific time or under certain conditions, represented in rule-based form. This enables contextual analysis focused on scenarios rather than relying solely on event-driven examination. Table 2 is an example of an integrated scenario in which customer personal information is decrypted and leaked via USB, and a method of derivation. It integrates scenarios in which a person who downloaded more than 100 customer information per day from the CRM, a personal information processing system, decrypted more than 100 of the documents, scenarios in which the USB writing PC security system was bypassed, and scenarios in which excessive access was made from a specific IP of the server system. In other words, through this, a customer personal information leak threat integrated scenario can be created.

**Figure 6:**
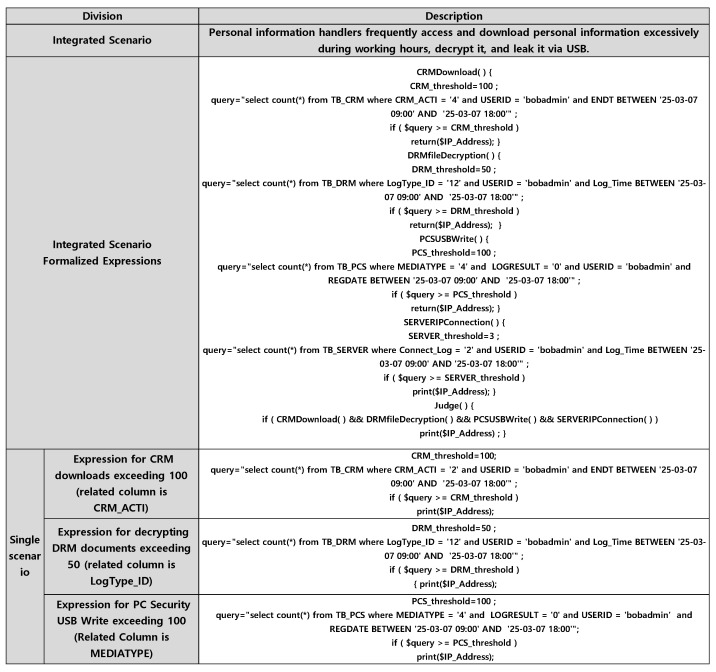
Standardized scenario expressions.

The derived integration and unit scenarios are expressed in a formalized form. Figure 6 formalizes the integration scenarios and their associated unit scenarios in Table 1.

We will try to standardize an integrated scenario in which a personal information handler named bobadmin frequently logs in and excessively downloads customer personal information, decrypts it, and leaks it via USB. In each scenario, if the personal information handler exceeds 100 CRM downloads during working hours on March 7, if the DRM document is decrypted more than 50 times, if the server system is accessed excessively more than 3 times, and if the number of PC security USB writes exceeds 100, the cases in which the threshold is exceeded are detected in the corresponding functions using if statements. The IP_Addresses that appear at this time are identified as factors that can be commonly associated and can be used as AND conditions in the judge() function to detect the leakage of personal information of the corresponding user.

**Figure 7:**
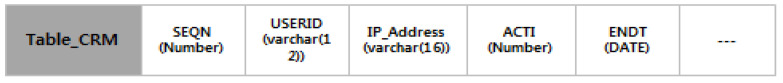
Linked CRM DB table structure.

To further understand the scenario of over 100 downloads of a standardized CRM system used in the above integration scenario, let us take a closer look at the Table schema. The related illustration is presented in Figure 7.

SEQN, ACTI are Number types, and IP_Address, USERID, etc., are varchar types to create a schema. The column that can control access to personal information files is ACTI. ACTI’s 1 is for selecting, 2 is for download, 3 is for modification, and 4 is for printing, and the code values are configured. Therefore, if CRM downloads exceed 100, ACTI = 2 corresponding to downloads can be set and extracted by executing the SQL Query Count below.

CRM_threshold = 100;

query = “select count(*) from TB_CRM where CRM_ACTI = ‘2’ and USERID = ‘bobadmin’ and ENDT BETWEEN ‘25-03-07 09:00’ AND ‘25-03-07 18:00’”;

if ($query >= CRM_threshold)

print ($IP_Address);

Threshold adjustment is performed quantitatively based on the frequency of log events. For example, if there were no anomalies in the previous month and the increase in downloads is due to regular business activities, the CRM threshold is adjusted dynamically. Specifically, the average number of CRM downloads over the month is calculated, and if this value exceeds the initial threshold of 100, the CRM threshold is updated to the new average. If the average does not exceed 100, the threshold remains unchanged. The initial value of 100 downloads was derived based on the daily average over a three-month period for the same site. The example below shows the raw code for threshold adjustment applied in April, based on the data from March.

default_threshold = 100; user_id = “bobadmin”

start_date = “25-03-01”; end_date = “25-03-31”

query_avg = “SELECT AVG(cnt) FROM (SELECT COUNT(*) AS cnt FROM TB_CRM WHERE CRM_ACTI = ‘2’ AND USERID = ‘${user_id}’ AND ENDT BETWEEN ‘${start_date} 09:00’ AND ‘${end_date} 18:00’ GROUP BY TO_CHAR(ENDT, ‘YYYY-MM-DD’)) AS daily_counts;”

avg_count = $(execute_sql “$query_avg”)

CRM_threshold = $default_threshold

if (($(echo “$avg_count > $default_threshold” | bc -l))); then

CRM_threshold = $avg_count

fi

today_start = “25-04-07 09:00”; today_end = “25-04-07 18:00”

query_today = “SELECT COUNT(*) FROM TB_CRM WHERE CRM_ACTI = ‘2’ AND USERID = ‘${user_id}’ AND ENDT BETWEEN ‘${today_start}’ AND ‘${today_end}’;”

count_today = $(execute_sql “$query_today”)

if (($(echo “$count_today >= $CRM_threshold” | bc -l))); then

print “$IP_Address”

fi

**Figure 8:**
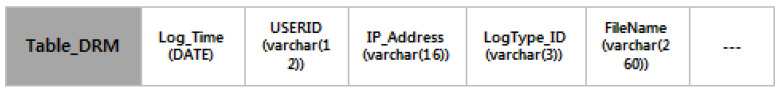
Linked DRM DB table structure.

The table schema related to the scenario of 50 decrypted documents are also shown in Figure 8.

Log_Time is of DATE type, and IP_Address, LogType_ID, FileName, etc., are of varchar type, and the schema is configured. Document decryption threats can be detected in the LogType_ID column. 12 of LogType_ID is configured as decryption, 13 is decryption failure, and 14 is Logout.

DRM_threshold = 50;

query = “select count(*) from TB_DRM where LogType_ID = ‘12’ and USERID = ‘bobadmin’ and Log_Time BETWEEN ‘25-03-07 09:00’ AND ‘25-03-07 18:00’”;

if ($query >= DRM_threshold)

print($IP_Address);

Therefore, the 50 cases decrypted in DRM can be processed with LogType_ID = 12, which is the decryption case, in the SQL Query Count above. Similarly, the standardized expression in Figure 5 can be confirmed in other server systems and security systems.

#### 3.2.4. Monitoring

The monitoring phase provides signs of abnormal behavior such as personal information leakage detected in the scenario. It shows summary information and detailed information on internal control violations by individual and organization and provides Top 5 and 10 statistical information by period such as daily, weekly, and monthly. It analyzes alerts judged to be normal work among the detected logs and adjusts the thresholds of the corresponding scenarios. This allows you to maintain and operate optimized scenarios for customers. In addition, it can secure monitoring visibility at the company level and increase the efficiency of responding to personal information leakage threats.

## 4. Empirical Results and Extension

### 4.1. Verification of the Privacy-Aware Integrated Log System Model

#### 4.1.1. Environment for Validating the Research Model

**Figure 9:**
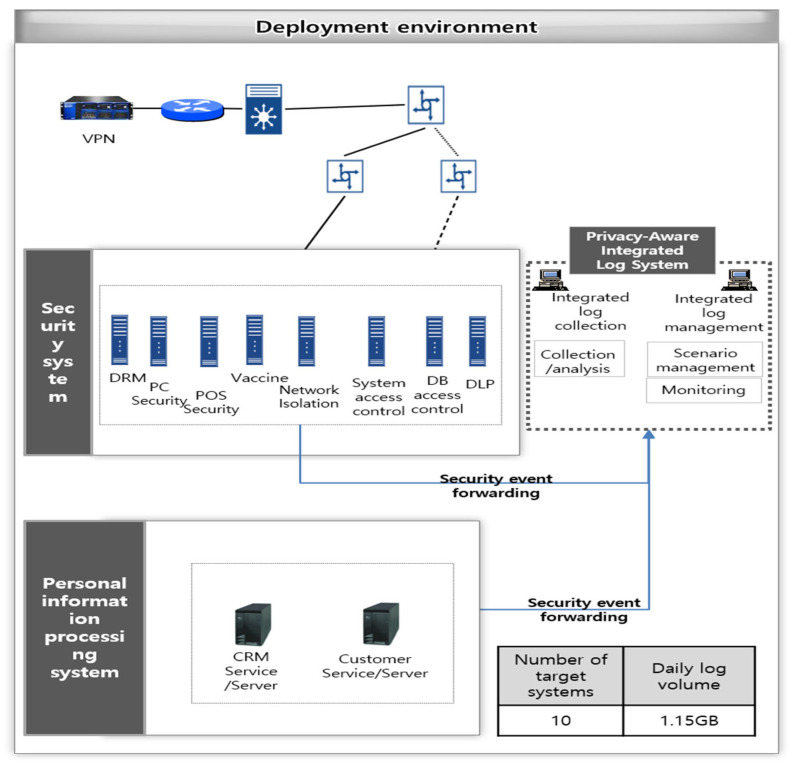
Deployment environment.

In this environment, internal employees were using separate networks, divided into a common network and a shopping mall network that performed external Internet business. To protect this, firewalls, IPS, DDoS, and VPNs for the common network for external remote access were operated on each network. In addition, POS terminals, which are IoT devices, were operated for customer payment on the separated network. Afterwards, document security that performs document encryption and decryption and integrated PC security that controls media such as USB were established as terminal security. Various security systems are being operated, such as server access control that controls access to major servers and DB access control systems for DB protection. Figure 9 illustrates the configuration diagram of a case study developed using the research model. In terms of log collection, logs were gathered from eight security systems such as DRM, PC security, and system access control, along with two systems handling personal data. The total daily log volume reached approximately 1.15 GB. Endpoint security solutions including DRM and PC security contributed a comparatively higher amount of log data. The scenario logs related to the phase immediately preceding resignation were triggered in approximately 3% of cases. Meanwhile, the objective of the application is to derive an integrated response scenario and to apply and validate it within a real operational environment.

If we look more closely at the linkage environment for this research model, we linked the logs of multiple internal control systems operating on PC terminals and business systems containing customer personal information. In particular, document decryption history information in document security, DB access control, and major table queries in personal information processing systems are important triggers for detecting customer personal information leaks. Additionally, the site acknowledges that collecting employee activity logs may pose potential risks of violating data protection regulations such as the GDPR and CCPA. To mitigate these risks, several safeguards have been implemented. First, the internal privacy policy document clearly stipulates the legal basis and purpose of log collection, thereby ensuring compliance and legitimacy. In line with the principle of privacy by design, a robust access control mechanism has been adopted to restrict system access exclusively to designated administrators. To prevent the collection of unnecessary information, data gathering is minimized. Biometric data is not collected, and sensitive log fields are protected through masking and encryption to address the risk of personal information leakage.

#### 4.1.2. Applying the Research Model

In order to integrate internal security systems, we first conducted a status analysis on the collection log analysis of events, etc., generated by the functions of the internal control systems of each unit in operation and the applied security policies. In particular, when designing a scenario rule considering the interrelationship of each internal security system for the detection of personal information leakage threats and then applying it to the research model system, if the log collection analysis of the internal control systems of each unit is not properly performed, event logs related to the scenario may not be generated. Analysis of target selection and linkage method. In order to collect events generated and stored in each internal control system into the research model system, we checked how each system stores its own event log and found the most efficient method to determine the linkage method. If the event is stored in a DB table, a view of the table is created and searched in batch form at a certain time to bring it into the Privacy-Aware Integrated Log System. This ensures security for table access and business availability. In cases where it cannot be brought in through DB search, it was confirmed that collecting events in the form of syslog, sftp, etc., is the most effective linkage method.

Table 3 shows the selected target systems for linkage, the main information collected from the system, and how the logs were linked. In addition, the settings and log creation of each selected system were confirmed in the analysis of the collected logs to collect valid logs. Table 4 shows the verification of whether each security policy was set in the main target systems for linkage to derive a valid scenario. From a log parsing perspective, in the case of database-based logs, relevant log data is directly retrieved from the associated database tables and utilized for analysis. In comparison, syslog-based logs are received via a syslog daemon. Each log format is then parsed into a structured JSON format before being stored in a unified log table. For these syslog messages, key fields such as timestamp, user, IP address, and event type are extracted. These values are subsequently normalized by mapping them to commonly used fields such as actor, action, asset, IP, and time. This structured mapping enables their use in integrated scenario analysis. To address the challenge of processing the growing volume of log data more efficiently, a slim log modeling approach is adopted. This approach enhances daily log processing capabilities by eliminating unnecessary fields and reducing overall storage demands. For instance, in logs generated by a DRM solution, encrypted input/output file size information was excluded, contributing to the reduction in storage overhead.

Accordingly, if a threat response scenario is created and applied to access an important system at dawn on Sunday, bring an important document to a PC, and decrypt the document, the corresponding scenario event does not occur because the access time by day of the week is not set for system access control. In addition, if an integrated scenario is created using the history log for the output of document security and the main server access history of the main system access control, the corresponding event does not occur. This is because the policy was not applied to document security and was applied to PC security, so the unit response scenario must be readjusted. In other words, the corresponding integrated scenario must be changed to a combination of PC security and system access control. In this way, the log collection and analysis for each unit’s internal control systems is very important.

Second, in the customer information system, which is a personal information protection processing system, a unit scenario that can be derived from 5W1H was created, and a threat scenario that can bypass internal security systems such as DRM and DB access control and a server system threat scenario were derived. In the personal information processing system, personal information such as phone numbers and account numbers was divided into the personal information handler, system operator, Blacklist criteria, and where aspects of internal and external networks. In addition, the How aspects of DB query, download, change, and printing were performed. In other words, various unit scenarios were derived using the 5W1H method.

**Figure 10:**
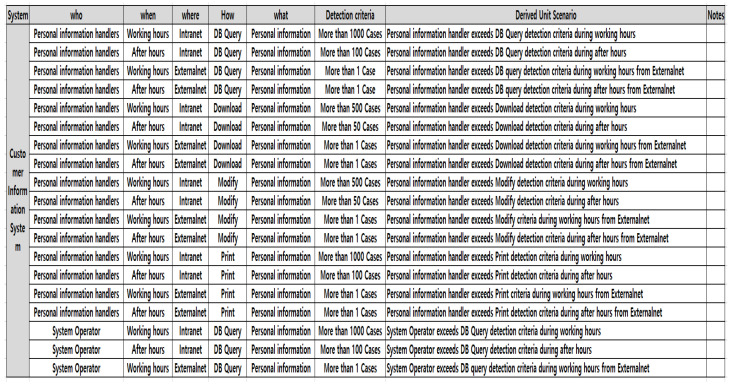
Derivation for personal information processing system threat scenario.

In particular, Figure 10 was able to derive various actions, such as threats such as personal information handlers downloading more than 500 customer information items from the personal information processing system during working hours. In addition, for DRM in operation to protect important documents, security system bypass unit scenarios were derived from the 5W1H perspectives such as administrator, decryption, logout count, and working hours.

Applicable scenarios were selected based on selection criteria such as validity, which determines whether they can be used as scenarios, and risk relevance, which indicates the degree of association with attack risk.

**Figure 11:**
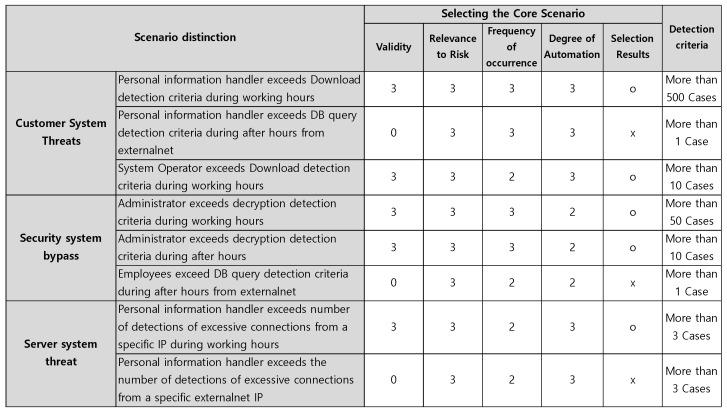
Selecting the core scenario.

Figure 11 shows examples of selected core scenarios. If the scenario exceeds the detection criteria for DB Query outside of working hours by a personal information handler, personal information handlers are not permitted to access the company outside of working hours, such as on holidays, in principle. In other words, the scenario was excluded from selection in consideration of the customer environment validity, which is a selection criterion for judging the value of the scenario.

Third, among the selected core scenarios, the optimal integrated scenarios were derived and applied by integrating the threat scenarios for personal information processing systems such as customer systems and personnel systems, the security system bypass response scenarios such as DRM, DLP, PC security, and DB access control, and the server system threat scenario.

**Figure 12:**
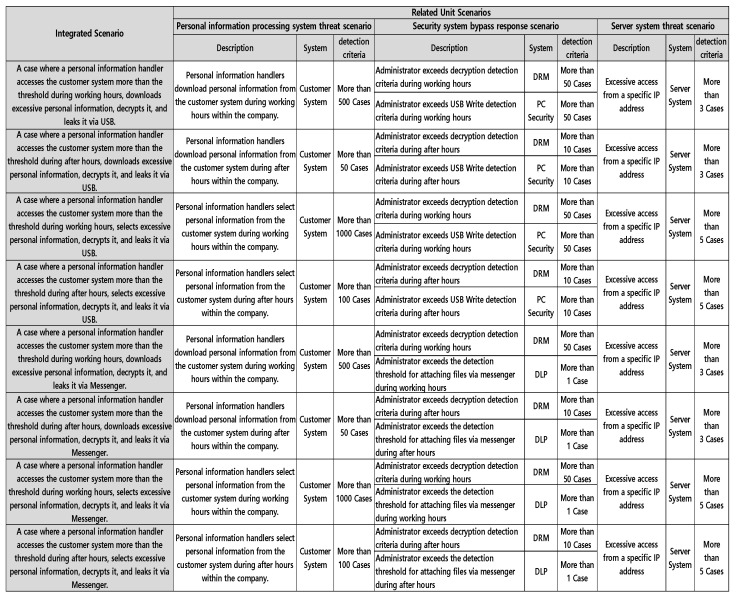
Derived integrated scenario case.

**Figure 13:**
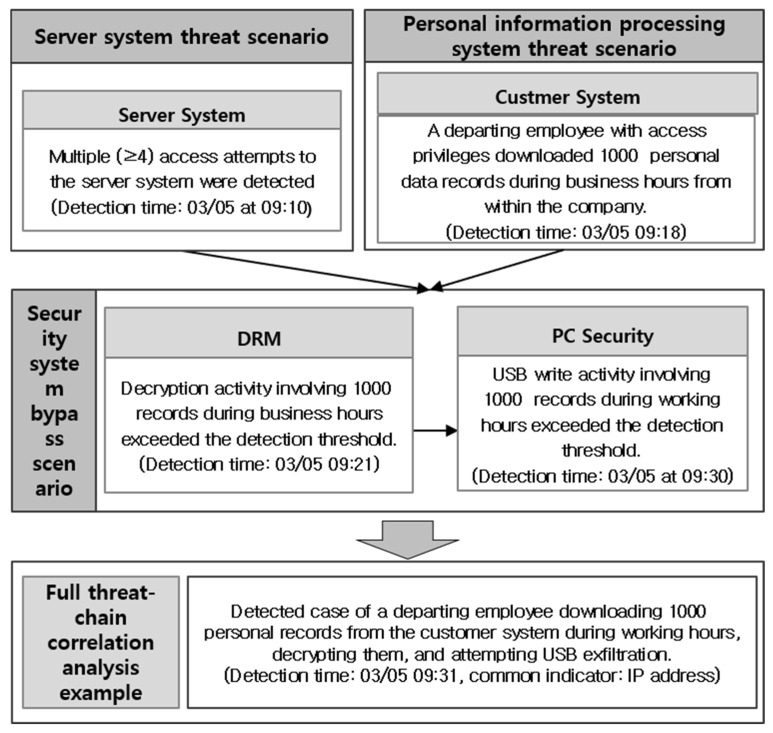
Full threat-chain correlation analysis example.

Figure 12 shows integrated scenario cases derived from the Privacy-Aware Integrated Log System model. In addition, the scenarios were analyzed by reflecting the latest attack response, customer personal information leakage patterns, and company IT culture. For example, internal employees had a high turnover rate due to the nature of the industry. Control over personnel was necessary due to the frequent retirement of employees compared to other industries. Therefore, a scenario was created that focused on managing those scheduled to retire, and through this, internal information leakage threats were responded to. In other words, the HR department periodically provided relevant information on high-risk personnel, such as those scheduled to retire, who needed to be managed intensively. The received relevant information was regularly batched into the Privacy-Aware Integrated Log System and updated to the relevant integrated scenario rules to continuously update the system. In this way, various scenarios were created through the Privacy-Aware Integrated Log System model, which derives integrated scenarios after linking events between internal security systems such as DRM, DLP, and PC security. Through this, it was verified that it is possible to respond more effectively to personal information leakage threats. Figure 13 below illustrates an actual log flow triggered by high-priority scenarios identified through correlation analysis of the overall threat chain.

**Figure 14:**
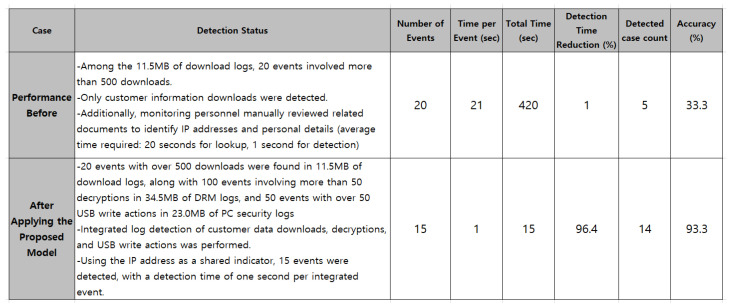
Numerical results before and after model application at the site.

As shown in Figure 13, multiple access attempts occurred at 09:10, followed by the download of 1000 files by 09:18. Between 09:20 and 09:30, decryption and USB write operations were attempted. The integrated detection scenario successfully identified this activity at 09:31. Additionally, Figure 14 presents the comparative results before and after applying the proposed model, confirming improvements in detection time and accuracy.

In terms of detection time, the integrated scenario detection using a common IP indicator reduced the time from 420 s to 15 s, achieving a 96.4% reduction. Simultaneously, detection accuracy significantly improved from 33.3% to 93.3%, demonstrating a substantial enhancement in detection performance. Among the 15 suspected data leakage cases, one was not detected. This particular case involved an attacker gradually downloading and decrypting data over the course of a week, effectively evading detection. This highlights the need for future countermeasures against such tactics. Overall, the multi-linked event analysis based on integrated scenarios contributed to improved operational efficiency by enhancing both detection speed and accuracy.

#### 4.1.3. Comparison of the Proposed Research Model with Existing Systems

**Figure 15:**
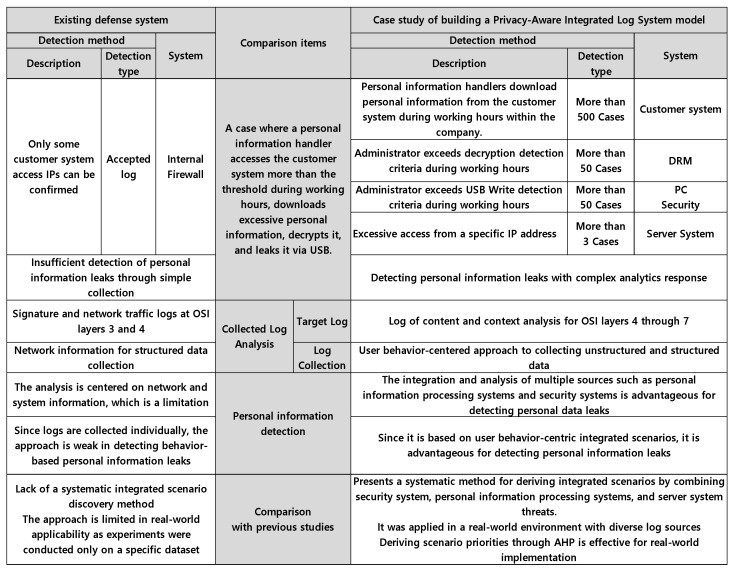
Comparison between the existing defense system and the proposed model.

In the past, security logs were collected and managed at the level of individual security devices, which led to structural limitations in performing correlation analysis across logs or detecting threats based on user behavioral patterns. These constraints presented challenges in recognizing and responding to complex security incidents, such as the leakage of personal information, through log interrelationships. This issue is attributed to detection approaches that focus solely on individual events. In this study, a Privacy-Aware Integrated Log System model based on users’ behavioral flow and multiple log sources was proposed to facilitate responses to customer information leakage threats. Integrated scenario analysis that collects events that occur in internal control systems, derives scenarios, and responds by linking them can be an active and three-dimensional response to increasingly sophisticated attacks. For example, this study has a method of responding by linking scenarios to actions that access a DB server, obtain personal information from a customer table, decrypt the document, and leak it to a USB. In other words, it was more effective because there were many event sources to analyze correlations and events for actions could be interconnected in context. Figure 15 shows that the model was built on the customer site and the superiority of the collected log analysis and scenario derivation compared to the existing one was confirmed, and this will enable more effective response to customer personal information leakage. In addition, the superiority of the model was verified by comparing it with existing related studies.

### 4.2. Extension Through AHP

In this study, in developing a scenario-based Privacy-Aware Integrated Log System model, AHP was extended to secure effectiveness and validity in an actual operating environment. AHP enables verification of whether the model has practicality and logic in various ways by numerically quantifying the relative importance of scenarios based on the judgment of a group of experts.

#### 4.2.1. Deriving Key Scenario Items

**Figure 16:**
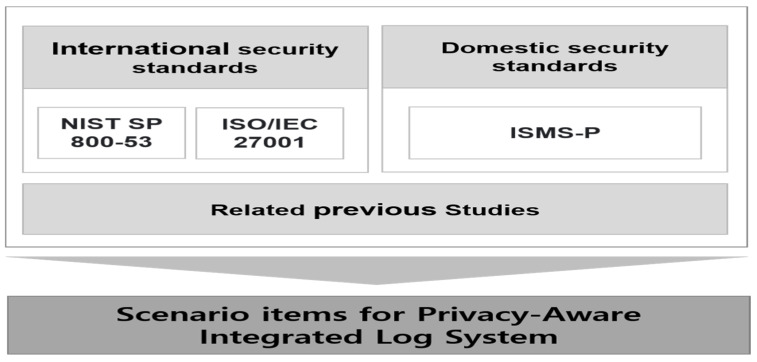
Deriving scenario items.

In addition to the theoretical considerations that have been studied previously, the scenario items of the Privacy-Aware Log Integration System model were derived from overseas security guides such as NIST SP 800-53 and ISO/IEC 27001, and Korea’s ISMS-P. The contents are as shown in Figure 16.

**Figure 17:**
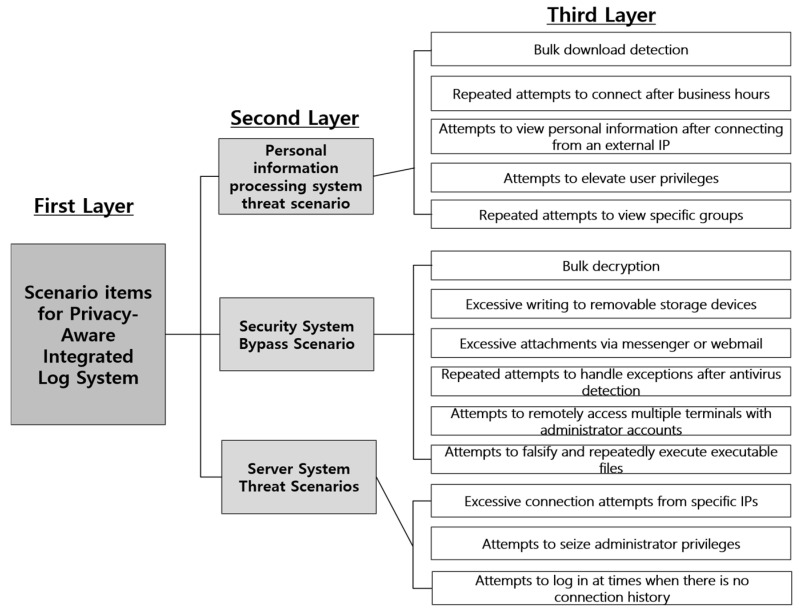
Schematic diagram for relative importance.

Based on this, the schematic diagram for evaluating the relative importance between items based on the main scenario items of the Privacy-Aware Integrated Log System model is as shown in Figure 17.

The goal was divided into the first tier, the second tier into three areas of the main scenario items: personal information processing system threat scenario, security system bypass scenario, and server system threat scenario, and the sub-evaluation criteria were organized. The third tier consisted of 14 sub-items, and the personal information processing system threat scenario items included mass download detection, repeated attempts to access after working hours, and attempts to view personal information after connecting an external IP. Security system bypass scenarios include mass decryption, excessive writing to removable storage devices, excessive attachments via messengers or webmail, repeated attempts to handle exceptions after antivirus detection, attempts to remotely access multiple terminals with administrator accounts, and attempts to falsify and repeatedly execute executable files. Lastly, server system threat scenarios include excessive attempts to connect from specific IPs and attempts to seize administrator privileges.

#### 4.2.2. Determining Relative Importance and Results

1. Survey method: An expert survey was conducted from March 11 to April 11, 2025. Initially, explanations for each item were provided via phone and social media to improve accuracy, followed by the collection of responses through email. To enhance objectivity, the evaluation framework was structured with three upper-level criteria and fourteen lower-level sub-criteria, and pairwise comparisons were performed within the same hierarchy. The evaluation employed the 9-point scale, which is considered the most accurate in AHP questionnaires. This scale is used to determine the relative importance between two items. The survey was conducted with 23 security experts, all of whom hold recognized professional certifications such as ISMS-P, CISSP, or CISA, and have more than five years of experience in the security field, both domestically and internationally. In particular, we set the Consistency Ratio threshold at 0.1, which is the statistically validated tolerance level established by Dr. Thomas L. Saaty, the developer of the AHP. While AHP is a method based on expert survey data, its consistency and objectivity are well recognized in the current academic literature. In this study, 21 experts showed CR values below the threshold of 0.1, indicating acceptable consistency. However, two experts presented CR values of 0.291 and 0.159, respectively, which exceed the threshold. Consequently, we excluded these two responses from the analysis. The age distribution showed that 52.4% of the respondents were in their 40s, representing the largest group. Regarding education level, over 57% held a master’s degree or higher. In terms of professional experience in the information security industry, 61.9% had between 11 and 19 years of service, making it the most prominent category. In terms of background distribution, 71.4% of the participants were from industry, while 28.6% had an academic background.
2. AHP procedure: To determine the priority of the Privacy-Aware Integrated Log System scenarios, the AHP methodology was applied in a step-by-step manner. In the first phase, scenario items were structured hierarchically. In the second phase, pairwise comparison surveys were conducted with security experts to evaluate the relative importance of each item. In the third phase, consistency ratios were examined, and the relative weights of each level were calculated using the pairwise comparison matrices. Finally, overall priorities were derived by aggregating the weights from both upper-level and lower-level items. Particularly in the third phase, a pairwise comparison matrix was constructed to determine the relative importance among the lower-level factors. The rows and columns of the matrix were defined by *m* and *n* criteria, respectively, and the matrix structure is presented in Equation (1).

(1) $$D_{mn}\;=\;\frac{1}{D_{nm}}$$

This expression reflects the reciprocal property, which is a fundamental axiom of AHP. Therefore, in the *D* matrix below, the element at the $D_{21}$ position is expressed as $1/D_{12}$. If item *m* is more important than item *n*, a score between 9 and 1 is assigned from the left. If *m* and *n* are of equal importance, the score is 0. If *m* is less important than *n*, a score between 1 and 9 is assigned from the right. In AHP, quantitative comparison of priorities is typically conducted using a defined interval scale such as −9 to 9, −7 to 7, or −5 to 5, depending on a consistent set of rules. To enable precise comparisons, a 9-point scale was employed to allow for finer differentiation in scoring. Accordingly, the resulting pairwise comparison matrix can be expressed as follows.

$$D=\left(\begin{array}{cccc} 1 & D_{12} & .. & D_{1n}\\ 1/D_{12} & 1 & .. & D_{2n}\\ : & : & : & :\\ 1/D_{1n} & 1/D_{2n} & .. & 1 \end{array}\right)$$

To derive the weight vector, the pairwise comparison matrix is multiplied.

$$DD=\left(\begin{array}{cccc} 1 & D_{12} & .. & D_{1n}\\ 1/D_{12} & 1 & .. & D_{2n}\\ : & : & : & :\\ 1/D_{1n} & 1/D_{2n} & .. & 1 \end{array}\right)\left(\begin{array}{cccc} 1 & D_{12} & .. & D_{1n}\\ 1/D_{12} & 1 & .. & D_{2n}\\ : & : & : & :\\ 1/D_{1n} & 1/D_{2n} & .. & 1 \end{array}\right)=\;\left(\begin{array}{ccc} b_{11} & .. & b_{1n}\\ \vdots & .. & \vdots \\ b_{m1} & .. & b_{mn} \end{array}\right)\;=B$$

It is defined as a *B* matrix. The row-wise sums are given below.

$$\begin{array}{l} E_{1}=b_{11}+b_{12}\ldots \;\ldots +b_{1n}=\sum_{n=1}b_{1n}\\ E_{2}=b_{21}+b_{22}\ldots \;\ldots +b_{2n}=\sum_{n=1}b_{2n}\\ \;\;\;\;\;\;\vdots \;\;\;\;\;\;\;\;\;\;\;\;\;\;\;\;\;\;\;\;\;\vdots \;\;\;\;\;\;\;\;\;\;\;\;\;\;\;\;\;\;\;\;\;\;\;\;\;\;\;\vdots \\ E_{m}=b_{m1}+b_{m2}\ldots \;\ldots +b_{mn}=\sum_{n=1}b_{mn} \end{array}$$

The sum of the entire matrix can be represented as follows.

$$B_{total}=\sum_{n,m=1}^{n,m}b_{mn}=E_{1}+E_{2}\ldots \;\ldots +E_{m}=\sum_{m=1}E_{m}$$

Accordingly, the weight vector value for the *j*-th row is given by Equation (2).

(2) $$w_{j}=\frac{E_{j}}{\sum_{m=1}E_{m}}$$

The weight vector for each row is calculated based on the product of the pairwise comparison matrix and Equation (2). Accordingly, when the weight vector for each row corresponding to the threat scenario items of the personal data processing system is calculated, the values are derived as 0.5200, 0.0770, 0.0350, and so on. Based on these weights, it can be determined that the most critical sub-areas include ‘Bulk download detection,’ ‘Repeated attempts to view specific groups,’ and others, ranked in descending order of importance.

3. Relative importance results and significance; The results of the relative importance analysis of the Privacy-Aware Integrated Log System scenario areas and detailed sub-items for responding to personal information leaks using AHP are as shown in Table 5.

This analysis provides the weighted values for each scenario item at the second layer, referred to as the upper level, and the third layer, referred to as the lower level. By integrating both layers, the overall relative priority of the detailed scenario items can be identified. According to the empirical results, the pairwise comparison among upper-level categories showed that the relative importance of the personal information processing system threat scenario, the security system bypass scenario, and the server system threat scenario was 47.9 percent, 45.8 percent, and 6.3 percent, respectively. Therefore, the personal information processing system threat scenario was found to be the most critical among the upper-level elements. The similar weighting of Security Bypass (45.8%) and Personal Information System Threat (47.9%) is interpreted as a reflection of realistic patterns observed in actual internal data leakage incidents, where these two risk factors often occur simultaneously or sequentially. For example, it is common for insiders to decrypt data or access it through bypass channels immediately after downloading large amounts of personal information. This suggests that both threats are structurally interconnected within a single act. The top 5 relative importance items derived through AHP analysis are quantitative judgment grounds that can improve the operational efficiency and early detection rate of the integrated log system, and support the practical validity of this study. In other words, if you select 5 items with relative importance out of 14 and develop and operate them based on the corresponding scenarios, you can detect personal information leakage threats early. In addition, when there are many detection logs and human resources are insufficient, we can improve the efficiency of operational personnel through the top 5 items. This study developed an integrated response scenario from the perspective of personal data protection, based on international security standards such as ISO/IEC 27001 and NIST SP 800-53, and verified its practical applicability through direct implementation in an actual operational environment. Additionally, by applying the AHP methodology, the study quantified assessments from multiple security experts to objectively prioritize scenarios. This approach aims to enable the effective allocation of limited security resources and achieve tangible improvements in response systems. By going beyond the simple repetition of standard procedures, the study presents a practical and optimized supplementary model tailored to real-world organizational environments.

## 5. Conclusions and Future Work

In response to the growing landscape of multilayered security threats, such as advanced attacks based on large language models, inter-state cyber warfare, and increasingly sophisticated ransomware attacks, this study proposes a Privacy-Aware Integrated Log System model focused on personal information. The goal is to overcome the limitations of conventional static security policies and single system-based response approaches. Emphasizing the recent rise in insider-related data breaches, the study highlights the need for a personal data-centric integrated log system that can analyze contextual information by correlating data across multiple systems and security logs. Accordingly, this study analyzed logs generated from personal information processing systems, security systems, and server systems, and it derived integrated threat scenarios based on the findings. Through ongoing monitoring, enhanced security visibility was achieved. Furthermore, the effectiveness of the proposed model was validated by applying it to a case study and comparing it with existing response systems. In addition, in the verification phase, the relative importance of key scenarios was quantitatively analyzed by applying AHP, and Bulk download detection and Bulk decryption ranked high. The analyzed priorities were able to identify key scenarios that require priority response in a realistic security operation environment, and they showed that they can be used as practical criteria for strategically allocating limited operation personnel and resources. In situations where there are many monitoring targets within a short time frame or limited time and resources, focusing operational efforts on the top-ranked scenario items identified by this study’s relative importance analysis can enable early detection and rapid response to personal data breaches. This approach can also enhance time and cost efficiency. Finally, this study presents both academic and practical value by proposing a scenario-based contextual integrated log system, rather than relying solely on event-driven detection. In particular, the analysis of the relative importance of scenario items can serve as a foundation for policy design and monitoring criteria in future automated security systems such as SIEM and SOAR.

In line with the current trend in which AI plays a central role across the security landscape, we plan to advance our system into a dynamic threat response framework by integrating an AI-based abnormal behavior log detection model with the existing system. In future research, we intend to utilize Spark and Elasticsearch to process logs on the terabyte scale and implement a multi-layered cache structure to validate computational efficiency and scalability. We will also conduct empirical experiments using real-world data to measure key performance indicators such as trigger frequency and detection accuracy and perform comparative evaluations against conventional methods. In addition, we aim to establish procedures that incorporate the latest threat trends, thereby preventing the omission or oversight of scenario items and ensuring their continuous relevance.

Meanwhile, during the empirical phase of this study, the AHP-based evaluation was conducted exclusively with security experts to ensure reliability. However, this introduced limitations in generalizing the relative importance rankings. Therefore, future work will expand and diversify the survey sample to include a broader range of stakeholder groups, allowing for a comparative analysis of how relative importance rankings differ across these groups.

## Figures and Tables

**Table 1:** Comparison of the previously mentioned standards.

Standard	Key Features	Perspective on Customer Data Protection	Relevance to This Study
ISO/IEC 27001	International standard for Information Security Management Systems	Focused on risk management and information security policies	Provides a comprehensive framework for privacy protection
NIST SP 800-53	U.S. federal framework for security and privacy controls	Detailed technical and managerial control elements	Includes control items useful for log collection, analysis, and behavior-based detection
ISMS-P	Korea’s integrated standard for information and personal data protection	Specifies protection requirements based on personal data flow and processing	Close association with mechanisms for responding to customer data breaches
GDPR	EU’s legal framework for personal data protection	Emphasizes consent-based processing, purpose limitation, data minimization, and user rights	Including justification for collecting logs related to employee behavior monitoring
Zero Trust Model	Security architecture based on “never trust, always verify” principle	Continuous authentication and authorization for users, devices, and applications	Applies security strategies such as least privilege and continuous monitoring to customer data access

**Table 2:** Examples of integrated scenarios and their derivation method.

Integrated Scenario		Associated Unit Scenarios							
	Personal Information Processing System Threat Scenarios			Security System Bypass Response Scenario			Server System Threat Scenarios		
	Description	System	Detection criteria	Description	System	Detection criteria	Description	System	Detection criteria
Personal data is accessed and downloaded in large volumes, decrypted, and exfiltrated through USB	Personal Data Download Count	CRM	1 day/100 cases	Number of documents decrypted	DRM	1 day/50 cases	Number of excessive connections	Linux	1 day/3 cases
				USB write count	PC Security	1 day/100 cases			
**Integrated Scenario** = Personal information processing system threat scenario ∧ Security system bypass response scenario ∧ Server system threat scenario									

‘∧’ indicates an ‘AND’ condition.

**Table 3:** Selecting target system and linking form.

Target System	Important Log Collection Information	Linked Form
DRM	Document Decryption History Screen Capture Event	DB
PC Security	USB write event History of print output	DB
POS Security	PE file change history Malware deletion event	syslog
Vaccine	Vaccine daemon off Patch Deploy history	DB
Network Isolation	Network Isolation Login History Inter-domain file transfer history	syslog
System access control	Telnet, ftp, etc., access history events Prohibition command execution events	DB
DB access control	DB access history event Executing commands such as selecting major tables	DB
DLP	External mail sending history External illegal messenger history	DB
Personal information processing system	Administrator access history information Administrator execution command history	DB
Server system	Excessive connection attempts Attempt to seize administrator privileges	rsyslog

**Table 4:** Analysis of logs collected from the linked system.

Distinction	Security Policy	Whether Logs Are Created
DRM	Authorize creators to edit, decrypt, etc. Force encryption on save and exit Block copy and paste Set watermarking for output Control document viewing counts	O O O X O
PC Security	USB Read and Write Control Mobile Phone Tethering Control Website Access Monitoring Print Watermarking and History Management Messenger Conversation History Monitoring	O O X O X
System access control	Account lock settings Prohibited command settings Connection IP and MAC settings Access service control such as ssh, sftp	O O O O
DLP	Control illegal sites Allow Webhard for specific groups Monitor messenger conversation history Monitor external transmission mail Control remote control service	O O O O O

**Table 5:** Relative importance analysis results for scenario items.

Second Layer		Third Layer			Final Relative Importance	Final Priorities
Items	Relative Importance of Second Layer	Scenario Items	Relative Importance of Third Layer	Priorities of Third Layer		
Personal information processing system threat scenario	0.479	Bulk download detection	0.5200	1	0.2491	1
		Repeated attempts to connect after business hours	0.0770	3	0.0369	7
		Attempts to view personal information after connecting from an external IP	0.0350	5	0.0168	12
		Attempts to elevate user privileges	0.0540	4	0.0259	9
		Repeated attempts to view specific groups	0.3140	2	0.1504	3
Security System Bypass Scenario	0.458	Bulk decryption	0.4240	1	0.1942	2
		Excessive writing to removable storage devices	0.2990	2	0.1369	4
		Excessive attachments via messenger or webmail	0.1080	3	0.0495	6
		Repeated attempts to handle exceptions after antivirus detection	0.0380	6	0.0174	11
		Attempts to remotely access multiple terminals with administrator accounts	0.0790	4	0.0362	8
		Attempts to falsify and repeatedly execute executable files	0.0530	5	0.0243	10
Physical level check	0.063	Excessive connection attempts from specific IPs	0.7980	1	0.0503	5
		Attempts to seize administrator privileges	0.1380	2	0.0087	13
		Attempts to log in at times when there is no connection history	0.0640	3	0.0040	14

## Data Availability

The original contributions presented in the study are included in the article; further inquiries can be directed to the corresponding author.

## Abbreviations

The following abbreviations are used in this manuscript:

AHP	Analytic Hierarchy Process
LLM	Large-scale Language Model
ESM	Enterprise Security Management
SIEM	Security Information and Event Management
IEC	International Electrotechnical Commission
ISO	International Organization for Standardization
